# Influence of Germanium Substitution on the Crystal Chemistry and Dielectric Properties of Mg_2_SnO_4_

**DOI:** 10.3390/ma18245557

**Published:** 2025-12-11

**Authors:** Yih-Chien Chen, Chun-Hsu Shen, Chung-Long Pan, Chun-Hao Tai

**Affiliations:** 1 Department of Electrical Engineering, Lunghwa University of Science and Technology, Taoyuan City 33306, Taiwan; ee049@mail.lhu.edu.tw (Y.-C.C.); watermelon87223@gmail.com (C.-H.T.); 2 Department of Electrical Engineering, Ming Chuan University, 5 De Ming Rd., Gui Shan District, Taoyuan City 33348, Taiwan; 3 Department of Electrical Engineering, I-Shou University, No. 1, Sec. 1, Syuecheng Rd., Dashu District, Kaohsiung City 84001, Taiwan

**Keywords:** dielectric properties, Mg_2_(Sn_1−x_Ge_x_)O_4_ ceramics, microstructure analysis

## Abstract

**Highlights:**

**What are the main findings?**

- Ge^4+^ substitution successfully forms a solid solution in Mg_2_SnO_4_ up to x = 0.03.
- Lattice contraction follows Vegard’s law, indicating stable Ge^4+^ incorporation.
- The optimal composition (x = 0.03, 1550 °C) shows ε_r_ = 8.0 and Qf = 67,000 GHz.

**What are the implications of the main findings?**

- Demonstrate that moderate Ge substitution improves dielectric performance.
- Provide structural insight into phonon scattering reduction in spinel ceramics.
- Offer design guidance for low-loss materials in microwave communication devices.

**Abstract:**

The effects of Ge^4+^ substitution on the microwave dielectric properties of inverse spinel Mg_2_SnO_4_ ceramics were systematically investigated. A series of Mg_2_(Sn_1−x_Ge_x_)O_4_ (x = 0.00–0.05) ceramics were synthesized via solid-state reaction and sintered at 1450–1600 °C. X-ray diffraction confirmed single-phase inverse spinel structures (Fd-3 m) for compositions up to x = 0.03, while minor MgSnO_3_ secondary phases appeared at x = 0.05. Rietveld refinement revealed a linear decrease in lattice parameter from 8.6579 Å (x = 0) to 8.6325 Å (x = 0.05), consistent with Vegard’s law for the substitution of smaller Ge^4+^ (0.53 Å, Shannon ionic radius, octahedral coordination) for Sn^4+^ (0.69 Å, Shannon ionic radius, octahedral coordination) in octahedral sites. Optimal dielectric properties were achieved at x = 0.03 sintered at 1550 °C; the dielectric constant (ε_r_) increased from 7.6 to 8.0, while the quality factor (Qf) improved by 19% from 56,200 to 67,000 GHz, which is attributed to reduced phonon scattering from Ge-induced lattice contraction. The temperature coefficient of resonant frequency (τ_f_) remained stable (−64 to −68 ppm/°C) across all compositions. Property degradation at x = 0.05 correlated with the onset of Ge^4+^ solubility limit and MgSnO_3_ formation. These results demonstrate that controlled Ge^4+^ substitution effectively enhances the microwave dielectric performance of Mg_2_SnO_4_ ceramics for communication applications.

## 1. Introduction

In recent years, research on microwave dielectric ceramics has increasingly focused on developing materials with enhanced performance characteristics, particularly aiming for high quality factors, improved temperature stability, and tailored dielectric constants. Advances have been made through the exploration of novel material compositions, microstructural engineering, and sophisticated fabrication techniques, driving the field towards the achievement of optimal performance in miniaturized electronic components used in wireless communication systems [1,2]. Specifically, efforts have been directed towards understanding the fundamental relationships between crystal structure, compositional modifications, and dielectric behavior [3].

Microwave dielectric ceramics play a crucial role in modern wireless communication technologies, particularly in applications that require miniaturized resonators, filters, and antennas. Mg_2_SnO_4_ ceramics have attracted significant research interest owing to their promising dielectric properties, including high quality factors (Qf), moderate dielectric constant (ε_r_), and low temperature coefficient of resonant frequency (τ_f_) [4,5]. These ceramics are advantageous due to their ability to maintain stable dielectric performance across a range of operational conditions, making them suitable for integration into complex electronic systems. However, enhancing the performance characteristics, especially the quality factor and thermal stability, remains an ongoing research challenge due to inherent limitations associated with structural imperfections and lattice dynamics [6,7,8].

One promising strategy to overcome these limitations is partial cation substitution at the B-site (Sn^4+^), where dopants can tune local structural environments and modulate phonon dynamics. Germanium (Ge^4+^) is a particularly attractive substitute due to its stable oxidation state and ionic radius (0.53 Å), which is significantly smaller than that of Sn^4+^ (0.69 Å). This size mismatch facilitates controlled lattice contraction, potentially reducing internal strain and enhancing dielectric performance without inducing secondary phase formation [9,10,11]. Recent studies have demonstrated that Ge^4+^ substitution plays a critical role in regulating the crystal structure and sintering behavior of various oxide ceramics. Specifically, Ge^4+^ incorporation alters lattice parameters, bond energy, and bond valence, thereby affecting both densification and dielectric response. The high polarizability of Ge^4+^ contributes to enhanced ε_r_. At the same time, improvements in Qf are associated with increased bond covalency, reduced oxygen vacancy concentration, and improved lattice symmetry—particularly in systems exhibiting hexagonal or spinel coordination frameworks. Moreover, Ge doping has been shown to suppress abnormal grain growth and promote uniform microstructure development, both of which are conducive to lower dielectric loss and higher Qf values [12,13,14,15].

Despite these findings, the effects of Ge^4+^ substitution in Mg_2_SnO_4_ remain largely unexplored. In particular, the influence of low-level Ge incorporation (x ≤ 0.05) on phase composition, microstructure, and dielectric performance under high-temperature sintering conditions has not been systematically investigated. This substitution is expected to enhance dielectric performance by reducing dielectric losses, improving temperature stability, and optimizing microstructural features. Furthermore, investigating varying levels of Ge substitution provides valuable insights into the correlation between lattice distortions, phase stability, and dielectric behavior. Detailed characterization using X-ray diffraction (XRD), Raman spectroscopy, scanning electron microscopy (SEM), and dielectric measurements is crucial for elucidating the underlying mechanisms driving property improvements [16]. Thus, investigating the effect of Ge substitution in Mg_2_SnO_4_ ceramics provides a promising pathway for achieving tailored and optimized dielectric properties, potentially opening avenues for broader applications in advanced microwave and millimeter-wave communication technologies.

## 2. Experimental Section

### 2.1. Sample Preparation

Mg_2_(Sn_1−x_Ge_x_)O_4_ (x = 0.00, 0.01, 0.03, 0.05) ceramics were synthesized via the conventional solid-state reaction method following the procedures reported in previous studies on Mg_2_SnO_4_-based spinel ceramics [4,16]. The starting materials included basic magnesium carbonate (4MgCO_3_·Mg(OH)_2_·4H_2_O, containing ~43.5 wt% MgO, analytical grade) as the magnesium source, and SnO_2_ (Showa (Buffalo, NY, USA), 99.9%) and GeO_2_ (Alfa (Ward Hill, MA, USA), 99.9%) as the tin and germanium sources, respectively. Stoichiometric amounts of raw powders were weighed according to the target formula and thoroughly mixed in ethanol using a planetary ball mill with zirconia media for 12 h to ensure homogeneity. The suspension was dried and sieved through a 200-mesh screen to break up agglomerates and ensure fine particle size distribution.

The mixed powders were pre-calcined at 1200 °C for 4 h in air, allowing for the decomposition of the magnesium precursor and the formation of the Mg_2_(Sn_1−x_Ge_x_)O_4_ phase. After calcination, the powders were ground again to improve particle packing and reactivity. Polyvinyl alcohol (PVA, 5 wt%) was added as a temporary binder, and the powders were uniaxially pressed into cylindrical pellets (~10 mm diameter, ~5 mm thickness) under a pressure of 2000 kg/cm^2^. The green pellets were sintered at various temperatures (1450–1600 °C) for 4 h in air to investigate the effect of sintering temperature on the phase composition and dielectric properties.

### 2.2. Characterization

Phase identification was conducted using an X-ray diffractometer (Rigaku D/MAX-2200, Rigaku Corporation, Tokyo, Japan) with Cu–Kα radiation (λ = 1.5406 Å), operated at 40 kV and 30 mA. The scans were collected over a 2θ range of 20–80° with a step size of 0.02° and a scan speed of 2°/min [17].

Microstructural observations were performed using field-emission scanning electron microscopy (FE-SEM, JEOL JSM-6500F, JEOL, Tokyo, Japan) at an accelerating voltage of 15 kV and a working distance of approximately 10 mm. The average grain size was determined using ImageJ (version 1.54, National Institutes of Health, Bethesda, MD, USA; available at: https://imagej.nih.gov/ij/; accessed on 1 October 2025) by measuring at least 50 grains from multiple SEM regions for each composition, covering an analyzed area of more than 5000 µm^2^. Grain sizes were obtained using the equivalent-circle-diameter method and are reported as mean ± standard deviation. Elemental analysis was performed using an energy-dispersive X-ray spectroscopy (EDS, Oxford Instruments, Oxford, UK) system attached to the FE-SEM, operated at 15 kV to verify the elemental distribution and compositional uniformity [18].

Raman spectra were acquired using a Renishaw inVia Reflex spectrometer (Renishaw plc, Wotton-under-Edge, UK) equipped with a 532 nm excitation laser, operated at 1% laser power (~0.5 mW). An 1800 g/mm diffraction grating and a 50× objective lens was used to collect the spectra. Each spectrum was recorded with an exposure time of 10 s and three accumulations to improve the signal-to-noise ratio [19].

The dielectric constant (ε_r_) was measured using the cavity perturbation method, while the quality factor (Qf) was determined in the TE_01_δ mode using a vector network analyzer (Keysight N5224A, Keysight Technologies, Santa Rosa, CA, USA) [20]. The temperature coefficient of resonant frequency (τ_f_) was determined by monitoring resonant frequency shifts over a temperature range of 25 °C to 85 °C [21,22,23].

## 3. Results and Discussion

**Figure 1:**
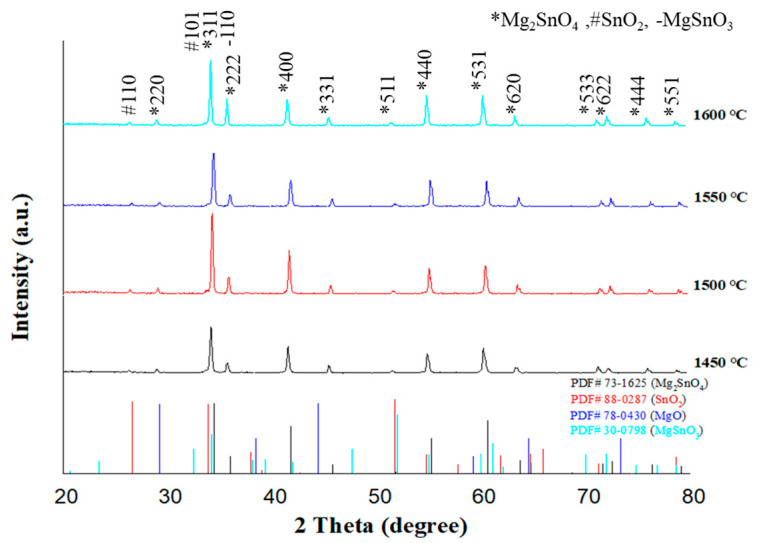
X-ray diffraction (XRD) patterns of Mg_2_(Sn_0.97_Ge_0.03_)O_4_ ceramics sintered at 1450–1600 °C for 4 h.

The X-ray diffraction (XRD) patterns shown in Figure 1 confirm that Mg_2_(Sn_0.97_Ge_0.03_)O_4_ ceramics, sintered at temperatures between 1450 °C and 1600 °C, predominantly exhibit the inverse spinel phase Mg_2_SnO_4_ (PDF# 73-1625). Minor secondary phases, including SnO_2_ (PDF# 88-0287) and MgSnO_3_ (PDF# 30-0798), are also present. The volume fraction of SnO_2_ was quantitatively estimated using an intensity ratio method, based on the integrated peak intensities of the (110) reflection of SnO_2_ and the (311) reflection of Mg_2_SnO_4_. This can be calculated using the equation

(1) $${SnO}_{2}\;\left(vol\%\right)=\frac{I_{100({SnO}_{2})}}{I_{311({Mg}_{2}{SnO}_{4})}+I_{100({SnO}_{2})}}\times 100\%$$

As shown in Table 1, complete phase purity (100% M.P.) is attained at 1600 °C for x = 0.01 and 0.05, suggesting that sintering above 1550 °C enhances Sn^4+^/Ge^4+^ solid solubility and suppresses secondary phase formation. These results are consistent with previous studies on Mg_2_SnO_4_-based systems, which have shown that high-temperature sintering (above 1500 °C) enhances crystallinity by promoting oxygen stoichiometry and cation ordering within the spinel lattice. The uncertainty in the estimation of the secondary SnO_2_ phase is approximately ±2–3 vol%, originating from peak fitting and background subtraction.

**Figure 2:**
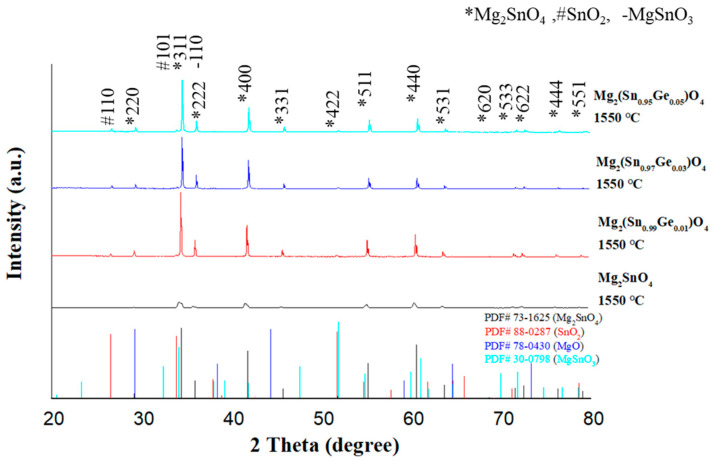
XRD patterns of Mg_2_(Sn_1−x_Ge_x_)O_4_ ceramics with different Ge substitution levels (x = 0.00–0.05) sintered at 1550 °C for 4 h.

The structural changes resulting from the substitution of Ge are clearly illustrated in Figure 2, which shows the X-ray diffraction patterns of Mg_2_(Sn_1−x_Ge_x_)O_4_ ceramics sintered at 1550 °C for 4 h. A systematic shift in the (*311) diffraction peak is observed from 34.25° at x = 0 to 34.64° (x = 0.01), 34.75° (x = 0.03), and 34.82° (x = 0.05). This progressive shift toward higher 2θ values confirms lattice contraction due to the substitution of smaller Ge^4+^ ions (0.53 Å) in place of larger Sn^4+^ ions (0.69 Å) within the Mg_2_SnO_4_ lattice. The nearly linear trend—averaging a peak shift of 0.194° per atomic percent of Ge—indicates the formation of a continuous solid solution. This behavior is consistent with Vegard’s law, which predicts a linear relationship between lattice parameters and compositional variation in crystalline solids.

The Rietveld refinement results, summarized in Table 2, confirm a clear reduction in the refined lattice parameter (a = b = c) from 8.6579 ± 0.0016 Å at x = 0 to 8.6325 ± 0.0008 Å at x = 0.05. This reflects the progressive substitution of larger Sn^4+^ ions by smaller Ge^4+^ ions within the Mg_2_SnO_4_ lattice. This trend is consistent across both the refined and calculated values, demonstrating the structural reliability of the synthesized solid solution. In addition, bond length analysis reveals a gradual increase in the Mg_1_–O_1_ bond length in the tetrahedral site, which goes from 1.8156 Å to 1.9886 Å. Concurrently, there is a reduction in the octahedral Mg_2_–O_1_ and Sn_1_–O_1_ bond lengths in the octahedral site, decreasing from 2.1881 ± 0.0005 Å at x = 0 to 2.0870 ± 0.0003 Å at x = 0.05. These changes indicate local structural rearrangements within the spinel lattice, reflecting the size mismatch and associated strain effects resulting from the incorporation of Ge. Furthermore, the weighted profile R-factor (R_wp_) ranges from 9.69% to 14.37%, indicating acceptable refinement quality for all compositions. The lowest R_wp_, observed at x = 0.03, implies a relatively more stable and homogeneous structure at this doping level. Overall, the data support the idea that Ge^4+^ substitution in Mg_2_SnO_4_ leads not only to lattice contraction but also to subtle modifications of the local coordination geometry, particularly within the octahedral sites. This finding aligns with observations in other spinel-type titanate and germinate ceramics, where cation substitution induces anisotropic distortion and affects structural stability [4,24].

**Figure 3:**
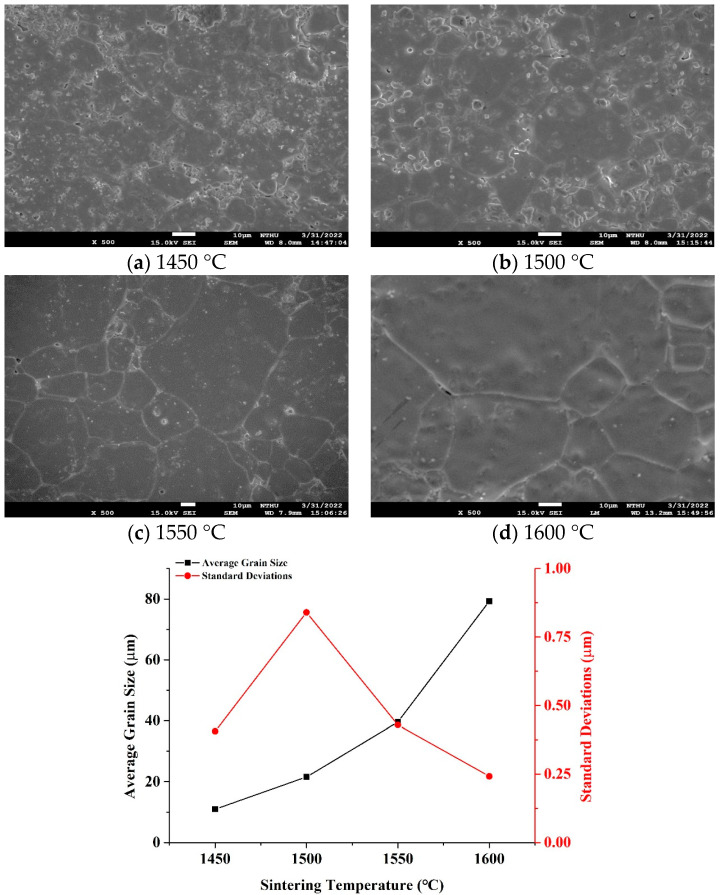
The SEM images and average grain size of Mg_2_(Sn_0.97_Ge_0.03_)O_4_ ceramics were obtained with various sintering temperatures for 4 h.

The SEM images in Figure 3 illustrate the microstructural evolution of Ge-substituted Mg_2_SnO_4_ ceramics (x = 0.03) sintered at various temperatures (1450–1600 °C). A clear grain growth trend is observed, with average grain size increasing markedly from 10.96 μm at 1450 °C to 79.27 μm at 1600 °C, as quantified in Figure 3. This behavior reflects enhanced atomic diffusion and grain boundary mobility at elevated temperatures, consistent with previous studies on Mg-based spinel ceramics. At 1450 °C, the microstructure consists of fine, closely packed grains with numerous intergranular pores, indicating limited sintering. As the temperature increases to 1500 °C, initial grain coalescence becomes evident, accompanied by moderate densification. At 1550 °C, grains exhibit well-defined boundaries and significantly reduced porosity, suggesting near-optimal densification. However, further increasing the temperature to 1600 °C results in abnormal grain growth, which could negatively impact mechanical integrity and promote microcracking due to thermal mismatch stresses.

**Figure 4:**
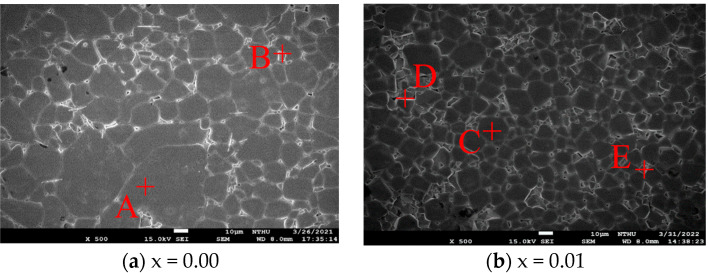

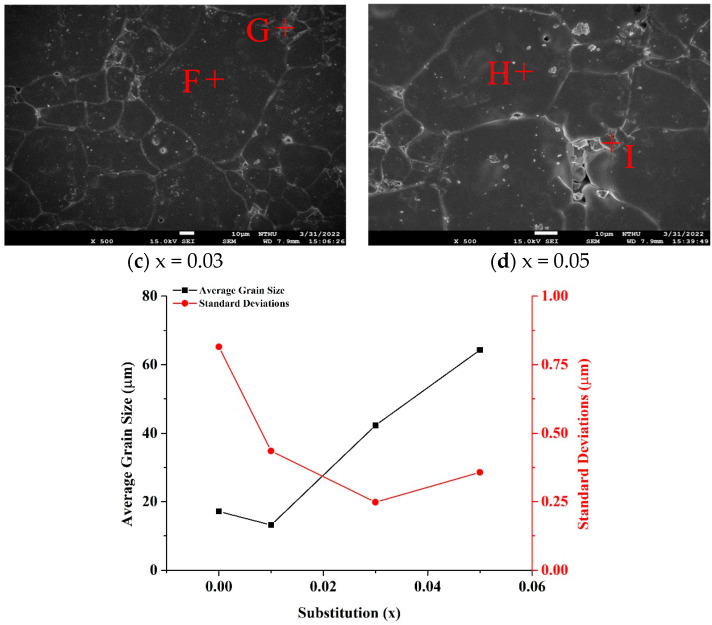
The SEM images and average grain size of Mg_2_(Sn_1−x_Ge_x_)O_4_ ceramics obtained with various x values sintered at 1550 °C for 4 h.

In addition to the sintering temperature, the level of germanium (Ge) substitution (denoted as x) significantly influences the behavior of grain growth. As illustrated in Figure 4, the average grain size increases progressively with increasing Ge content. This trend indicates that the incorporation of Ge enhances grain boundary diffusion, promoting grain coarsening. This effect is likely due to the lower diffusion energy activation of Ge^4+^ compared to Sn^4+^, which facilitates mass transport during sintering and accelerates the kinetics of grain growth. For compositions with x values ranging from 0.01 to 0.05, a consistent increase in grain size is observed, with x = 0.05 showing the largest grains. EDS data presented in Table 3 reveal more uniform elemental distributions at x = 0.03 and 0.05. This finding suggests improved phase stability and a reduction in the formation of secondary phases at higher Ge levels. However, trace amounts of MgSnO_3_ phases are detected at x = 0.05, indicating that the solubility limit for Ge may be approached, which could lead to phase segregation. The elemental quantification provided by EDS also verifies that the ratios of Mg:Sn:Ge:O closely match the nominal stoichiometry of Mg_2_(Sn_1−x_Ge_x_)O_4_, confirming the successful incorporation of Ge into the spinel lattice. While moderate Ge substitution improves microstructural homogeneity and suppresses the formation of secondary phases, excessive Ge content (specifically at x = 0.05) may induce phase instability and the formation of MgSnO_3_, potentially harming dielectric performance.

**Figure 5:**
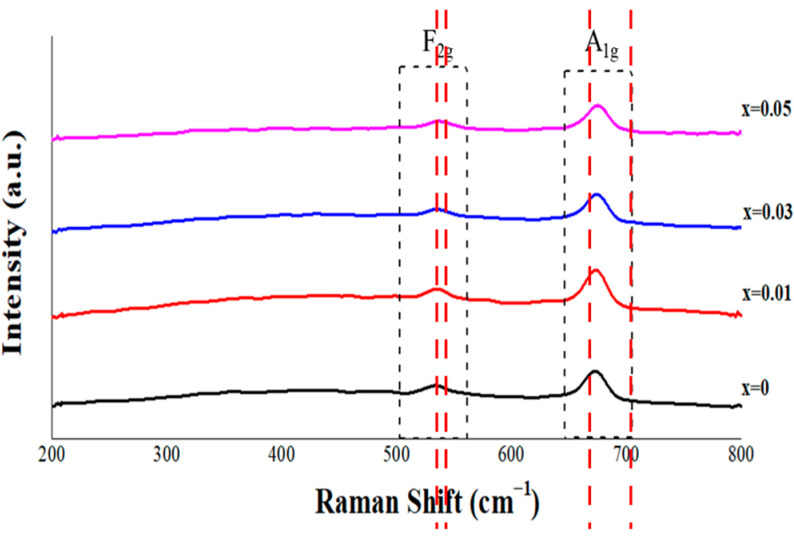
Raman spectra of Mg_2_(Sn_1−x_Ge_x_)O_4_ ceramics (x = 0–0.05) sintered at 1550 °C for 4 h, showing compositional effects on the F_2g_ and A_1g_ vibrational modes. Vertical red dashed lines are added to highlight the compositional shifts in these modes.

Raman spectroscopy was employed to investigate the vibrational modes and local structural changes in Ge-substituted Mg_2_SnO_4_ ceramics (x = 0–0.05), as shown in Figure 5. All compositions displayed distinct Raman features corresponding to the F_2g_ and A_1g_ modes, which are characteristic of the inverse spinel structure. The F_2g_ mode, found around 530–537 cm^−1^, can be attributed to the translational vibrations of Mg and Sn/Ge cations in tetrahedral and octahedral coordination. Meanwhile, the A_1g_ modes, located near 643 cm^−1^ and 674 cm^−1^, are associated with the symmetric stretching of oxygen atoms in octahedral sites. As the Ge content increased (from x = 0 to 0.05), a slight but consistent blue shift in the F_2g_ and A_1g_ peaks toward higher wavenumbers was observed. This blue shift indicates a reduction in bond lengths and an increase in bond strength, which is consistent with lattice contraction due to the smaller ionic radius of Ge^4+^ (0.53 Å) compared to Sn^4+^ (0.69 Å). Additionally, moderate peak broadening, especially in the F_2g_ region, suggests increased local structural disorder and microstrain resulting from cation substitution [24]. These spectral changes reinforce the conclusions drawn from XRD and Rietveld refinement, confirming that the substitution of Ge modifies the local bonding environment and vibrational dynamics of the spinel lattice. These vibrational characteristics, including the F_2g_ (~530–537 cm^−1^) and A_1g_ (~643 and ~674 cm^−1^) modes as well as their blue-shift behavior, are consistent with previously reported Raman studies on spinel-type titanate and germanate ceramics [24,25]. The evolving Raman response indicates both enhanced structural compactness and slight increases in disorder.

**Figure 6:**
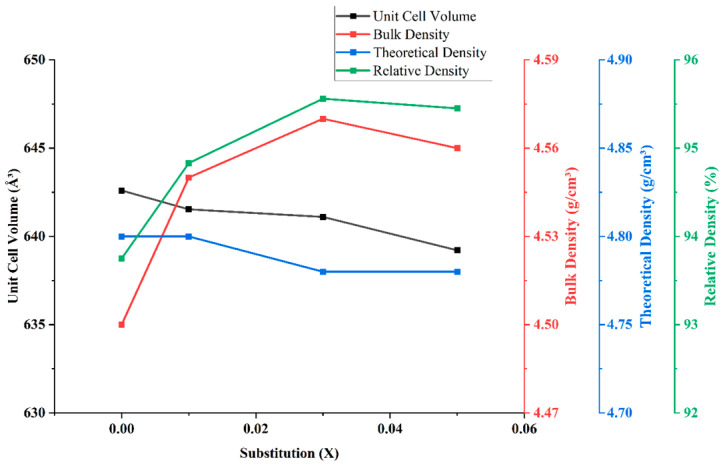
Variation in unit cell volume, bulk density, theoretical density, and relative density with Ge substitution (x) in Mg_2_(Sn_1−x_Ge_x_)O_4_ ceramics.

The unit cell volume, bulk density, theoretical density, and relative density (Dr) of Mg_2_(Sn_1−x_Ge_x_)O_4_ ceramics sintered at 1550 °C as a function of Ge content (x = 0.00–0.05) are illustrated in Figure 6. The unit cell volume shows a non-linear variation: it initially increases slightly at x = 0.01, but then gradually decreases for x = 0.03 and x = 0.05. This behavior reflects the competing effects of Ge^4+^ substitution and potential local lattice distortions. Although Ge^4+^ has a smaller ionic radius (0.53 Å) compared to Sn^4+^ (0.69 Å), its initial substitution may locally disrupt the lattice, resulting in a slight expansion of the lattice. However, at higher concentrations, overall lattice contraction becomes more dominant [25]. In terms of densification, both bulk density and theoretical density increase with Ge incorporation, especially at x = 0.01 and x = 0.03. This suggests enhanced atomic diffusion and improved sintering behavior, likely due to increased grain boundary mobility facilitated by the substitution of Ge. The relative density reaches a maximum of approximately 96% at x = 0.03, indicating optimal densification. However, at x = 0.05, a slight decrease in relative density is observed, which may be attributed to excessive grain growth and the formation of isolated pores, as confirmed by the SEM micrographs in Figure 4. These microstructural changes can hinder further densification and potentially affect the dielectric properties of ceramics.

**Figure 7:**
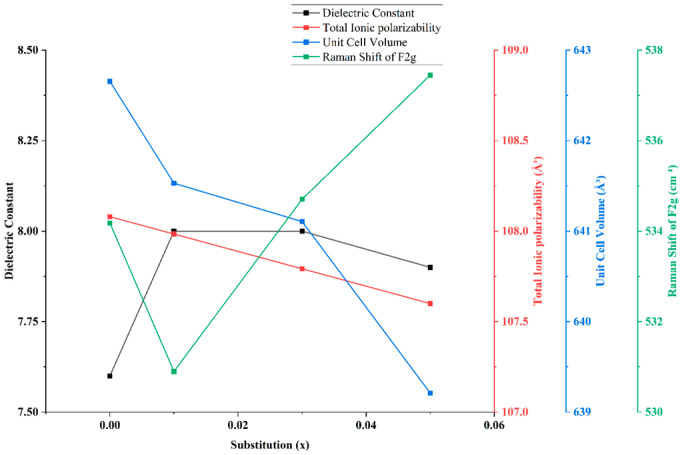
Correlation between dielectric constant, total ionic polarizability, unit cell volume, and Raman shift of F_2g_ mode with Ge substitution (x) in Mg_2_(Sn_1−x_Ge_x_)O_4_ ceramics.

Figure 7 illustrates the correlation among the dielectric constant (ε_r_), total ionic polarizability, unit cell volume, and Raman shift in the F_2g_ mode in Mg_2_(Sn_1−x_Ge_x_)O_4_ ceramics. The dielectric constant exhibits a non-monotonic trend, reaching a maximum at x = 0.01, followed by a gradual decrease at higher Ge concentrations (x = 0.03 and 0.05). This behavior is closely associated with structural and vibrational changes induced by Ge substitution. The decrease in ε_r_ beyond x = 0.01 correlates with a reduction in unit cell volume and a blue shift in the F_2g_ Raman mode. These observations suggest increased bond stiffness and reduced lattice polarizability, as Ge^4+^ substitution leads to shorter Mg–O and Sn/Ge–O bonds, which limit ionic displacement under an applied electric field. The blue shift in the F_2g_ mode further implies strengthened force constants and reduced lattice anharmonicity, both of which contribute to the observed decline in dielectric response [26]. In addition, the trend of total ionic polarizability closely mirrors that of ε_r_, underscoring its crucial role in determining dielectric permittivity. As Ge content increases, the decrease in polarizability—resulting from stronger and shorter bonds—reduces the ability of ions to respond to the electric field, thereby lowering ε_r_. These findings confirm that both structural modifications and intrinsic ionic polarizability cooperatively govern the dielectric behavior of Ge-substituted Mg_2_SnO_4_ ceramics [27].

**Figure 8:**
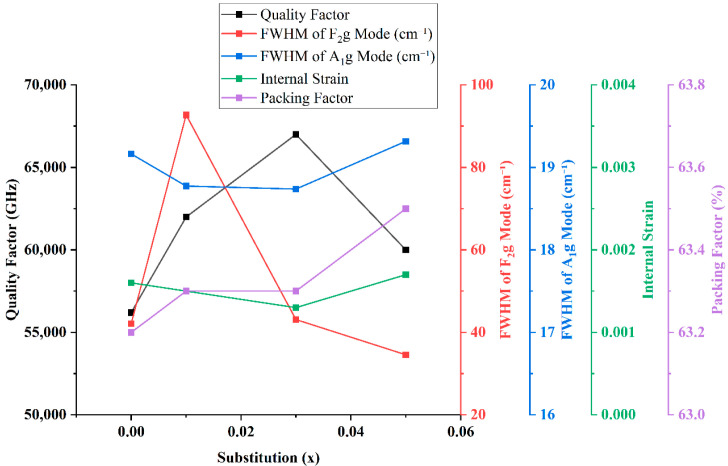
Correlation between the quality factor (Qf), the FWHM of the F_2g_ and A_1g_ modes, internal strain, and packing factor with Ge substitution (x) in Mg_2_(Sn_1−x_Ge_x_)O_4_ ceramics sintered at 1550 °C. The A_1g_ mode in this figure corresponds to the main Raman peak at ~674 cm^−1^.

Figure 8 illustrates the intricate relationship between the microwave dielectric quality factor (Qf) and the structural evolution of Mg_2_(Sn_1−x_Ge_x_)O_4_ ceramics sintered at 1550 °C. The Qf value increases substantially from 56,200 GHz at x = 0 to a peak of 67,000 GHz at x = 0.03, followed by a moderate decline to 60,000 GHz at x = 0.05. This behavior is strongly influenced by phonon scattering dynamics, as reflected by the full width at half maximum (FWHM) of the F_2g_ Raman mode. While the FWHM initially broadens significantly at x = 0.01 (92.7 cm^−1^), it narrows to 43.1 cm^−1^ at x = 0.03—coinciding with the highest Qf value—indicating reduced lattice anharmonicity and improved local vibrational coherence [28,29,30]. Interestingly, at x = 0.05, Qf decreases despite a further narrowing of the F_2g_ mode to 34.5 cm^−1^. This apparent contradiction suggests the influence of additional structural factors. Specifically, internal strain increases slightly from 0.0013 to 0.0017 between x = 0.03 and x = 0.05, implying a resurgence of lattice distortion. Moreover, EDS detects localized regions with a MgSnO_3_-type composition, indicating the presence of a minor secondary phase that is not captured in the XRD analysis. These compositional inhomogeneities likely introduce dielectric loss pathways such as defect-related phonon scattering or grain boundary disruptions. The packing factor increases marginally with x, from 63.2% at x = 0 to 63.5% at x = 0.05, reflecting improved densification across the composition range. However, this parameter shows a weaker correlation with Qf compared to vibrational and strain-related metrics. Overall, the optimal Qf at x = 0.03 arises from a synergistic balance of reduced phonon scattering, minimal internal strain, and high structural homogeneity—highlighting the efficacy of moderate Ge substitution in tuning lattice dynamics for enhanced microwave dielectric performance [31].

**Figure 9:**
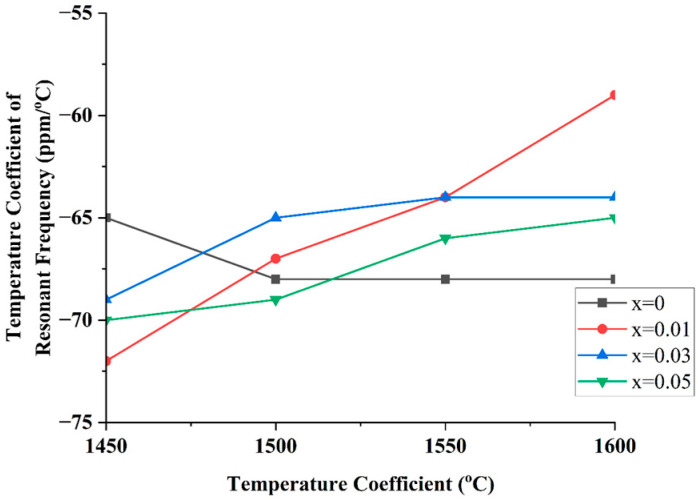
Variation in the temperature coefficient of resonant frequency (τ_f_) with sintering temperature for different Ge substitution levels (x) in Mg_2_(Sn_1−x_Ge_x_)O_4_ ceramics.

Figure 9 illustrates the evolution of the temperature coefficient of resonant frequency (*τ_f_*) in Mg_2_(Sn_1−x_Ge_x_)O_4_ ceramics as a function of sintering temperature. For all compositions, *τ_f_* values become less negative with increasing sintering temperature, indicating enhanced densification and improved compositional homogeneity. At lower Ge contents (x = 0 and 0.01), *τ_f_* remains relatively stable across the temperature range, suggesting minimal influence on the lattice’s thermal expansion behavior. In contrast, compositions with higher Ge substitution (x = 0.03 and 0.05) exhibit a more pronounced shift in *τ_f_* toward zero as sintering temperature increases. This trend suggests that Ge incorporation modifies the thermal expansion mismatch within the crystal lattice, likely due to unit cell contraction and increased bond stiffness associated with the smaller ionic radius of Ge^4+^. The observed *τ_f_* behavior correlates closely with changes in unit cell volume and the blue shift in Raman modes, indicating that resonant frequency temperature stability is governed by both structural distortion and vibrational dynamics.

## 4. Conclusions

This study demonstrated that Ge substitution in Mg_2_(Sn_1−x_Ge_x_)O_4_ ceramics effectively enhances their structural, dielectric, and thermal properties. All specimens were sintered at 1550 °C for 4 h, under which the incorporation of Ge^4+^ (0.53 Å) in place of Sn^4+^ (0.69 Å) led to controlled lattice contraction, improved densification, and a reduction in secondary phase formation. As summarized in Table 4, the dielectric constant (ε_r_) increased from 7.6 at x = 0.00 to 8.0 in the range of x = 0.01–0.03, which is attributed to enhanced ionic polarizability and microstructural compactness. A slight decrease at x = 0.05 is likely associated with grain coarsening and localized compositional deviations. The quality factor (Qf) exhibited a substantial improvement, increasing from 56,200 GHz (x = 0.00) to a maximum of 67,000 GHz at x = 0.03, primarily due to reduced phonon scattering, minimal internal strain, and enhanced phase uniformity. The temperature coefficient of resonant frequency (τ_f_) improved from −68 ppm/°C at x = 0.00 to −64 ppm/°C at x = 0.01–0.03, indicating better thermal expansion compatibility. A slight shift to −66 ppm/°C at x = 0.05 may result from residual strain and subtle structural perturbations. These findings confirm that the composition at x = 0.03, sintered at 1550 °C, achieves the most favorable balance of high relative density, enhanced dielectric constant, maximum Qf, and stable τ_f_, making it the optimal formulation for microwave dielectric and resonator applications.

## Figures and Tables

**Table 1:** Phase composition analysis, showing the percentage of main phase (M.P.) and secondary phase (S.P.) at various Ge substitution levels and sintering temperatures.

x Value	S.T. (°C)	M.P. (vol%)	S.P. (vol%)
0	1450	-	-
	1500	-	-
	1550	94.9	5.1
	1600	-	-
0.01	1450	94.1	5.9
	1500	93.0	7.0
	1550	96.9	3.1
	1600	100	0
0.03	1450	96.2	3.8
	1500	96.3	3.8
	1550	95.3	4.7
	1600	96.6	3.4
0.05	1450	96.7	3.3
	1500	100	0
	1550	100	0
	1600	100	0

Note: The uncertainty in the phase quantification is estimated to be ±2–3 vol%, mainly arising from peak fitting and background subtraction.

**Table 2:** Structural parameters of Mg_2_(Sn_1−x_Ge_x_)O_4_ from Rietveld refinement: lattice constants, bond lengths, and refinement quality indicators.

Parameters	x = 0	x = 0.01	x = 0.03	x = 0.05
Lattice parameter, a (Å)				
Refined value	8.6579	8.6379	8.6410	8.6325
Calculated value	8.6579	8.6379	8.6410	8.6325
Bond length (Å)				
Mg_1_–O_1_ (Å) (Tetrahedral)	1.8156	1.8756	1.9043	1.9886
Mg_2_–O_1_/Sn_1_–O_1_ (Å) (Octahedral)	2.1881	2.1109	2.1062	2.087
The reliability factor of weighted patterns: R_wp_ (%)	11.64	13.41	9.69	14.37

**Table 3:** EDS results of selected particles in Mg_2_(Sn_1−x_Ge_x_)O_4_ ceramics sintered at 1550 °C for 4 h.

	Spot	Mg	Sn	Ge	O	Mg:Sn:Ge:O
(a)	A	27.7	20.8	0.0	51.4	3:2:0:5 (Mg_2_SnO_4_)
	B	11.1	28.8	0.0	60.1	1:3:0:6 (SnO_2_)
(b)	C	29.3	17.2	0.7	52.8	3:1.7:0:5.3 (Mg_2_SnO_4_)
	D	16.5	18.5	0.5	64.5	2:2:0:6 (MgSnO_3_)
	E	9.4	44.4	0.1	46.1	1:4.5:0:4.5 (SnO_2_)
(c)	F	29.7	16.4	0.3	53.6	3:1.5:0:5.5 (Mg_2_SnO_4_)
	G	7.9	36.8	1.2	54.1	1:3.5:0:5.5 (SnO_2_)
(d)	H	31.0	28.3	1.8	38.9	3:3:0:4 (MgSnO_3_)
	I	29.7	15.1	1.2	54.0	3:1.5:0:5.5 (Mg_2_SnO_4_)

**Table 4:** Dielectric properties of Mg_2_(Sn_1−x_Ge_x_)O_4_ ceramics with various x value sintered at 1550 °C for 4 h.

x Value	Bulk Density (g/cm^3^)	D_R_ (%)	ε_r_	Qf (GHz)	τ_f_ (ppm/°C)
0.0	4.5	93.75	7.6	56,200	−68
0.01	4.55	94.83	8.0	62,000	−64
0.03	4.57	95.56	8.0	67,000	−64
0.05	4.56	95.45	7.9	60,000	−66

## Data Availability

The original contributions presented in this study are included in the article. Further inquiries can be directed to the corresponding authors.
